# A Real Medical Strike

**Published:** 1899-09-09

**Authors:** 


### The Hospital

A Journal of

The Medical Sciences and Hospital Administration.

Vol. XXVI.—No. 676.] Edited by Sir Henry Burdett, K.C.B., and Solomon C. Smith, M.D., M.R.C.P. [September 9, 1899.

### A Real Medical Strike.

We have often heard of strikes among medical men, which, however, have generally been but half-hearted affairs. The doctors have indeed struck, if refusing to work for the remuneration offered could be called striking, but the means taken to enforce a higher standard of payment have been, from the trades-unionist point of view, absolutely contemptible.

Now, however, we have before us the spectacle of a real strike, in which not only does a doctor here and there refuse to accept certain club fees, but in which all the medical men in the district combine to refuse them; in which not only do these medical men refuse to attend all clubs which decline the "union rate," but refuse to attend their members as private patients except at a special tariff; and in which, on the clubs endeavouring to gain their ends by introducing strangers—"blacklegs" as they would be called by trades-unionists—the medical men of the union have combined to refuse to attend any members of such clubs, even as private patients, whatever accident may befall them. They will in case of urgent danger to life give first aid, but then they will stand aside and the clubites must manage for themselves with such "blackleg" assistance as they can obtain.

This is what is now going on in Lancashire. The whole thing sounds to us very horrible, and it is only with a severe wrench to our professional instincts, and to all the modes of thought to which we have been accustomed, that we can think of members of our profession having to take up such a position. But such lamentable events are, after all, but the inevitable consequences of contract medical service. At this topsy-turvy end of the century all our older conceptions are apt to be reversed. In such a case as the one we are referring to the workmen are the employers—the masters, if they like the word—and the medical practitioners are the employed; and, curiously enough, in this reversed position we find an almost exact repetition of the old relations which, from the days when Charlotte Brontë depicted the events associated with the introduction of steam power into the manufacturing districts down to the present time, have always characterised conflicts between employers and the unorganised employed. It always has happened that, so long as the employed acted as individuals, they were pitted one against the other to beat down wages; and that is exactly what has happened in the "battle of the clubs," where organised societies have become the employers of medical men whose professional instincts have held them back from entering into trade combinations. Again, it always has happened that in the early days of trades unions the employers have endeavoured to break them down by the introduction of free labour; and that is exactly what has happened, again and again, in regard to club practice. Lastly, it always has happened that, in dealing with "blacklegs," things have been done which have been most regrettable—things contrary to the nature of the men themselves, and often such as no one of the men, acting for himself, would have ever thought of doing to his fellow; and we are afraid that here again we see a parallel in what is now happening in Lancashire.

The worst of it is that these things seem inevitable in trade disputes. In every trade and in every profession there are men who for various reasons are willing to work for lower remuneration than their fellows. Their reasons are often good ones, and to themselves seem absolutely convincing, and of such men "blacklegs" are made. But if a trades union is to raise the average wage, these men must either be forced to join the union and demand the union wages, or they must in some way be suppressed. This is a hard necessity, but it is fundamental. No trades union ever did or ever will exercise a really controlling influence over wages in the presence of free labour unless it can invent ways of rendering the employment of such labour difficult and not worth while. Hence many things are done which it is difficult to condone, and we are afraid that if medical men are bent upon a strike they also will have to do many things which, however little contrary to morals they may be, will be sadly contrary to professional traditions. Not that we blame them. Far from it. Their livelihood is being attacked by combinations which can only be met by counter combination. Still it is a pity, and it is all the more a pity since it seems to be the almost necessary outcome of a method of payment for professional attendance which is peculiarly fitted for those who receive a weekly wage.

The provident system of payment ought to solve many social problems for the working classes. But the way in which it has been worked in such places as Cork and Coventry, in Leicester, and now in Lancashire, is enough to make us doubt whether the evils associated with it are not greater than any advantages which it gives. We are quite clear that the provident system will lead to nothing but degradation of the profession unless the whole organisation is kept in the hands of the medical men themselves. No middlemen should be allowed to exploit our services. If the management is once given over to outsiders, whether these be friendly societies, or provident dispensary committees, or shop clubs, or medical aid associations, the tendency must inevitably be, as it always has been, to beat down the medical fees, and to encourage bad work. Against such evils the doctors can only protect themselves by the formation of trades unions with all their degrading but necessary accompaniments. What is happening in Lancashire is but the necessary and logical result of contract medical practice as it is ordinarily carried on, and this ought to be sufficient to condemn the system.

